# *Notes from the Field:* Two Cases of Legionnaires’ Disease in Newborns After Water Births — Arizona, 2016

**DOI:** 10.15585/mmwr.mm6622a4

**Published:** 2017-06-09

**Authors:** Geoffrey Granseth, Rachana Bhattarai, Tammy Sylvester, Siru Prasai, Eugene Livar

**Affiliations:** ^1^Arizona Department of Health Services; ^2^CDC/CSTE Applied Epidemiology Fellowship Program; ^3^Maricopa County Department of Public Health, Arizona.

Legionnaires’ disease is a severe, sometimes fatal disease characterized by fever, myalgia, cough, and clinical or radiographic pneumonia, caused by inhaling or aspirating small droplets of water containing *Legionella* bacteria.* In 2015, approximately 6,000 cases of Legionnaires’ disease were reported in the United States (*1*). Nearly 10% of cases are fatal (*2*). The number of reported cases of Legionnaires’ disease in Arizona has increased in recent years. Surveillance data from Arizona’s Medical Electronic Disease Surveillance Intelligence System (MEDSIS) identified 46 reported cases in 2011 and 93 in 2015 (*3*), representing more than a 100% increase. During 2011–2015, only one case was reported in an infant aged <1 month; however, during the first 4 months of 2016, two cases were reported in infants, both of whom were delivered at home in a birthing tub (water births).

The first case was reported to the Maricopa County Department of Public Health (MCDPH) during January 2016. The infant was delivered at home by a midwife on January 6, 2016 in a tub filled with tap water. The 1- and 5-minute Apgar scores were 5/10 and 9/10, respectively. The following day the infant was taken to a local emergency department with severe respiratory distress, tachypnea, and hypoxemia, where a diagnosis of congenital heart disease was made; the infant was transferred to children’s hospital A. An initial chest radiograph showed a confluent opacity in the lower left lobe, which was initially thought to represent atelectasis, although pneumonia could not be excluded. During the hospital stay, serial chest radiograph revealed persisting bilateral pulmonary infiltrates with possible cavitary lesions. The infant was later transferred to children’s hospital B where a bronchoscopy was performed, and a bronchoalveolar lavage culture tested positive for *Legionella pneumophila*, later identified at CDC as serogroup 1. The patient was treated with a 10-day course of azithromycin, but remained hospitalized for more than 2 months, primarily because of the congenital heart disease.

MCDPH conducted an epidemiologic investigation to identify the etiology of Legionnaires’ disease and provide recommendations based on potentially remediable transmission routes. The investigation revealed that a newly purchased birthing tub had been cleaned with vinegar and water before being filled with municipal tap water using a new drinking water hose immediately before the delivery. The mother delivered the child within an hour of entering the tub, and no aspiration by the infant was noted. No other risk factors for *Legionella* transmission were identified.

The second case was reported to MCDPH on April 18, 2016. The infant had been delivered by water birth at home on April 5 by a different, independently operating midwife, at home. 3 days after delivery, the infant developed a fever reported to be as high as 101.0°F (38.3°C); the fever recurred the following day, at which time the baby was brought to the emergency department of hospital A for evaluation; the infant’s temperature was 102.6°F (39.2°C) and a chest radiograph showed fluffy nodular opacities. The infant was admitted for treatment of neonatal sepsis and suspected pneumonia. On April 12, upper respiratory tract secretions and a urine specimen were collected. The urinary antigen test was positive for *Legionella pneumophila* antigen, and culture of the respiratory tract secretions was positive for *Legionella pneumophila*, later identified at CDC as serogroup 6.^†^ The patient was started on a 10-day course of azithromycin and later discharged on April 16.

An infection preventionist at hospital A familiar with the first case reported the second case to MCDPH after inquiring about the delivery method and learning of the home water birth. Investigation of this case revealed that the water birth had taken place in a rented jetted Jacuzzi hot tub. The tub had been filled with municipal tap water using a newly purchased hose and maintained at 98.0°F (36.7°C) in the bedroom for a week before the delivery. During the birth, the mother labored outside the tub and entered the tub for delivery only. No aspiration by the infant was noted.

Investigation of these two cases identified numerous gaps in infection prevention for water births, including use of a jetted Jacuzzi rather than a disposable birthing tub, and allowing the water to remain for a week at 98.0°F (36.7°C), which is within the optimum range for *Legionella* growth 77.0°F–108.0°F (25.0°C–42.2°C). Although the tub for delivery in the first case was filled immediately before the birth, tap water is not sterile, and *Legionella* can grow and spread in man-made water systems, such as plumbing systems. Because both tubs were emptied immediately after the births, no environmental sampling was performed.

During the follow-up investigation, a report of a Legionellosis death in an infant after a water birth in Texas in 2014 was identified (*4*). On the basis of subsequent guidelines developed by the Texas Department of State Health Services to assist licensed midwives conducting water births, the Arizona Department of Health Services (ADHS) and MCDPH, with support and guidance of the Arizona Healthcare-Associated Infection and Midwife Advisory Committees, developed educational resources and guidelines in November 2016.^§^^,^^¶^ These resources aim to increase knowledge about the risk for *Legionella* infection and maximize the safety for women choosing water immersion for labor or birth by providing a review of information on labor and birth in water. For example, although the risk for *Legionella* infection cannot be eliminated because of the need for warm tap water to fill the tub, it can be reduced by running hot water through the hose for 3 minutes before filling the tub to clear the hose and pipes of stagnant water and sediment. These materials have been distributed to a listserv of >1,300 Healthcare-Associated Infection contacts, shared with the local Association for Professionals in Infection Control and Epidemiology Chapter, and disseminated to all licensed midwives in the state. The materials are public and posted on the ADHS website at http://www.azdhs.gov/licensing/special/midwives/index.php#training.

## Footnotes

*
https://www.cdc.gov/legionella/about/index.html .

† The urine antigen is only designed to detect serogroup 1; however, cross reactions with other serogroups have been documented.

§
http://www.azdhs.gov/documents/licensing/special/midwives/training/guidelines-for-water-immersion-water-birth.pdf .

¶
http://www.azdhs.gov/documents/licensing/special/midwives/training/legionella-infographic.pdf .
